# Back to school – The teachers’ worries and needs having a childhood cancer patient or survivor in their class

**DOI:** 10.3389/fonc.2022.992584

**Published:** 2022-11-02

**Authors:** Maria Otth, Katrin Scheinemann

**Affiliations:** ^1^ Division of Oncology-Haematology, Department of Paediatrics, Kantonsspital Aarau, Aarau, Switzerland; ^2^ Department of Oncology, Haematology, Immunology, Stem Cell Transplantation and Somatic Gene Therapy, University Children’s Hospital Zurich, Zurich, Switzerland; ^3^ Department of Health Sciences and Medicine, University of Lucerne, Lucerne, Switzerland; ^4^ Department of Pediatrics, McMaster Children’s Hospital and McMaster University, Hamilton, ON, Canada

**Keywords:** pediatric oncology, school, teacher, women in science, reintegration, quality of life

## Abstract

**Background:**

A cancer diagnosis during childhood or adolescence causes nursery and school absences to various degrees. Attending school and meeting classmates gives many children and adolescents some normality back. Nevertheless, it can cause fears and concerns among the teachers. We are currently lacking information about the fears and needs of teachers having a child or adolescent diagnosed with cancer or with a cancer history in their classes. With this study, we aim to close this knowledge gap and assess the teachers’ fears, worries and information needs having a child or adolescent diagnosed with cancer in the class to develop a suitable information tool (flyer).

**Methods:**

We performed an online survey including teachers covering all grades from nursery to vocational school within the catchment area of our hospital. The survey included separate questions for experience with students still receiving active treatment and those in follow-up care. Answer options included tick boxes and open-ended questions, which we grouped thematically. We used descriptive analysis to describe the survey findings, resulting in a newly developed flyer.

**Results:**

In total 358 teachers participated in the survey, 80% were female, 63% worked in nursery or primary school. One quarter (26%) had experience with a student diagnosed with cancer. Most teachers with (81%) and without (85%) experience reported at least one concern. The top three concerns reported were: (1) how to inform the class, (2) the resilience of the student and (3) how to deal with the student and his or her family. The teachers preferred oral information by physicians or parents and written information equally. Information on resilience, guidelines with an emergency situation, and the need for cancer-specific information were considered important by about 75-94% of the teachers.

**Conclusion:**

Most teachers reported concerns, which we cover in a newly developed information flyer. However, such a flyer cannot replace individual communication between health care professionals and teachers. The identified concerns are likely to be transferable to other school systems and countries.

## Introduction

Education is an important cornerstone in a person’s life. Educational achievements determine to a significant extent the future professional life. It further influences a persons’ self-confidence, independence and the position in society (1, 2). A cancer diagnosis in children and adolescents often has a negative impact on school attendance and performance and may also alter or disrupt the relationship with peers and friends (3, 4). Diagnostic procedures, treatments and clinical visits often result in recurrent school absences of various durations. In addition, in treatment phases with a heavily compromised immune system school attendance is often not permitted by the treating physicians. Despite these necessary restrictions of regular school attendance, the goal is, that children and adolescents can participate at school as normally and frequent as possible. As shown by Tsimicalis et al, returning to school was perceived as very positive by the children and adolescents, gave some normality back and allowed to reconnect with peers (3). However, returning to school can be challenging for intellectual and social reasons (5, 6). Therefore, different school reintegration programs and support systems have been evaluated (7–9). For a successful school reintegration, teachers play a crucial role. Therefore, they need to be well informed and feel comfortable having an affected child or adolescent in their class. Literature and information on the potential fears, needs and uncertainties of teachers dealing with such a situation is currently missing. With this study, we aim to close this knowledge gap by describing the teachers’ fears, needs and uncertainties and by providing information material based on our findings. The final goal is to make reintegration of childhood cancer patients and survivors easier by taking the teachers’ needs into account.

## Methods

We performed a cross-sectional, questionnaire-based survey including teachers from nursery to vocational school, working in the catchment area of our pediatric oncology center (canton of Aargau and parts of canton of Solothurn, population around 800’000). We approached the principals from elementary schools in the canton of Aargau through the cantonal authority (Kantonaler Lehrerverband), searched the remaining schools on public websites and approached their principals directly. The principals were responsible to distribute the information letter and the link to our survey to all teachers of their school.

We developed the online survey for the purpose of this study (**Supplemental Explanation E1**). Answer options included tick boxes and open-ended questions, which we grouped thematically. The survey differentiated between teachers’ concerns, fears and information needs having a student newly diagnosed with cancer or a cancer survivor who completed treatment already. The survey had further separate sections for teachers with and without experience of having a childhood cancer patient or survivor in their class. We performed a pilot phase of the survey with three teachers from different school levels. They gave feedback on the surveys’ comprehensibility and structure. Following the implementation of their feedbacks, we distributed the information letter and the survey link, using SurveyMonkey^®^. We mainly performed descriptive analysis and used t-test and chi squared test to compare characteristics of different groups of teachers. For these analyses, we used STATA 17.0 and p-values <0.05 were considered statistically significant.

## Results

We approached 338 schools or principals and received feedback from 417 teachers. We excluded 59 teachers, who only completed the part on personal characteristics, but skipped all questions on fears and concerns. The characteristics of these teachers did not differ from those who completed the whole survey (**Supplemental Table S1**). Most teachers were female (80%) and worked in primary school levels (63%). Half of the teachers (51%) were the principal teachers of a class. The median time of working experience was 18 years (Interquartile Range, IQR 8 – 25) (**Table 1**).

**Table 1:** Characteristics of participating teachers (n=358).

	Number (%)
**Sex** Female	288 (80)
**Age** 18 – 34 years 35 – 44 years 45 – 54 years 55 – 64 years 65 years or older	83 (23) 81 (23) 100 (28) 90 (25) 4 (1)
**Working years** [years] Median (IQR)	18 (8 – 25)
School level* Primary level Secondary level I Secondary level II	226 (63) 100 (28) 147 (40)
**Role** Principal teacher of a class Individual school subjects Other	181 (51) 119 (33) 58 (16)
**Region** Rural Urban	192 (54) 166 (46)
**Experience** Yes No	94 (26) 264 (74)
**Brochure helpful** Yes No Missing	264 (74) 77 (21) 17 (5)

*more than one level possible per teacher; explanation in **Supplementary Explanation E1**.

One quarter of the teachers (n=94; 26%) reported having experience with a child or adolescent newly diagnosed with cancer or a childhood cancer survivor. Of those, 43 teachers reported that they received specific information. They received the information mainly from the parents (58%) or a combination of parents and health care professionals (19%) (**Table 2**). The main topics were about the students’ resilience (84%), the specific type of cancer (70%) and possible emergency situations for the child (35%). The information was sufficient for most teachers (72%) and was mainly delivered orally by parents (79%) or health care professionals, including physicians or hospital teachers (21%) (**Table 2**).

**Table 2:** Information received or needed stratified by teachers’ experience with children diagnosed with cancer.

	Number (%)
Teachers who had a child diagnosed with cancer in their class and received information (n = 43)	
**What was the situation of the child?** New diagnosis Treatment finished Missing	34 (79) 8 (19) 1 (2)
**What was the source of information?** Hospital Parents Hospital and parents Other Student him-/herself Another teacher Myself Missing	2 (5) 25 (58) 8 (19) 7 (16) 4 2 1 1 (2)
**What type of information did you receive?** Brochure from hospital Oral by physician, incl. hospital visit, hospital school Parents Link to homepage Other Student him-/herself Another teacher	5 (12) 9 (21) 34 (79) 0 6 (14) 2 4
**Which information did you receive?** Cancer in general Cancer specific Who to call in case of an emergency Possible emergency situations Student resilience	10 (23) 30 (70) 18 (42) 15 (35) 36 (84)
**Was the format of information appropriate?** Yes No* Missing	35 (81) 4 (9) 2 (5)
**Was the information sufficient?** Yes No° Missing	31 (72) 7 (16) 2 (5)
**Teachers who did not have a child diagnosed with cancer in their class or had a child in their class bur did not receive information (n=315)**	
**What would be the preferred source of information?** (multiple answer options) Flyer Physicians Parents Other^‡^	205 (65) 188 (60) 224 (71) 51 (16)
**Which information would be important?** (multiple answer options) Cancer general Cancer specific Who to call in case of an emergency Possible emergency situations Student resilience Other (all covered in the other survey sections)	142 (45) 235 (75) 108 (34) 275 (87) 297 (94) 54 (17)

* written information would have been better (n=3); only information from student (n=1).

° missing background knowledge (n=1); regular updates (n=2); contact point for questions (n=3).

^‡^ Child/adolescent and/or parents (n=19); school principal (n=8); specific contact point (n=5); hospital school, information event, webpage, round table (n=3 each); by parents and physicians together, childhood cancer Switzerland (n=2 each); hospital visit, video, children’s book (n=1 each).

Further 315 teachers did not have a child or adolescent diagnosed with cancer in their class or they had one in their class, but did not receive information. These teachers would prefer receiving information from parents (71%), written information material (65%) or orally by health care professionals (60%) (**Table 2**). As for teachers with experience with childhood cancer patients or survivors, the three most relevant topics would be on students’ resilience (95%), possible emergencies (87%) and the specific type of cancer (75%) (**Table 2**).

The fears and concerns mentioned by the teachers resulted in seven thematical groups: 1) students’ resilience; 2) dealing with the student and his or her family; 3) dealing with the topic of relapse and death; 4) possible emergencies; 5) infections and hygienic measures at school; 6) informing and dealing with classmates; 7) teachers’ own feelings (**Table 3**). No teacher reported any concerns related to potential legal responsibilities.

**Table 3:** Fears and concerns mentioned by the teachers, sorted in seven thematical groups (examples only).

Students’ resilience	«what can I ask from the child?»; «not to under- or over-challenge the child»; «physical and mental resilience»
Dealing with the student and its family	«respect for communication with the child»; «how can I help properly»; «how to deal with the child in everyday life»
Dealing with the topics of relapse and death	«dealing with the topic of death»; «I would have to think carefully how to integrate the topic of health, life, future perspectives and death in the lessons»
Possible emergencies	«how do I react correctly in case of an emergency»
Infections and hygienic measures at school	«risk of infectious diseases that could endanger the child»; «information on hygienic measures»
Informing and dealing with classmates	«how and what to communicate to the class»; «reaction of classmates»
Teacher	«how do I deal correctly with such a situation as a teacher»; «fear to find the correct words»; «fear of the unknown»; «respect of not behaving correctly»

**Figure 1:**
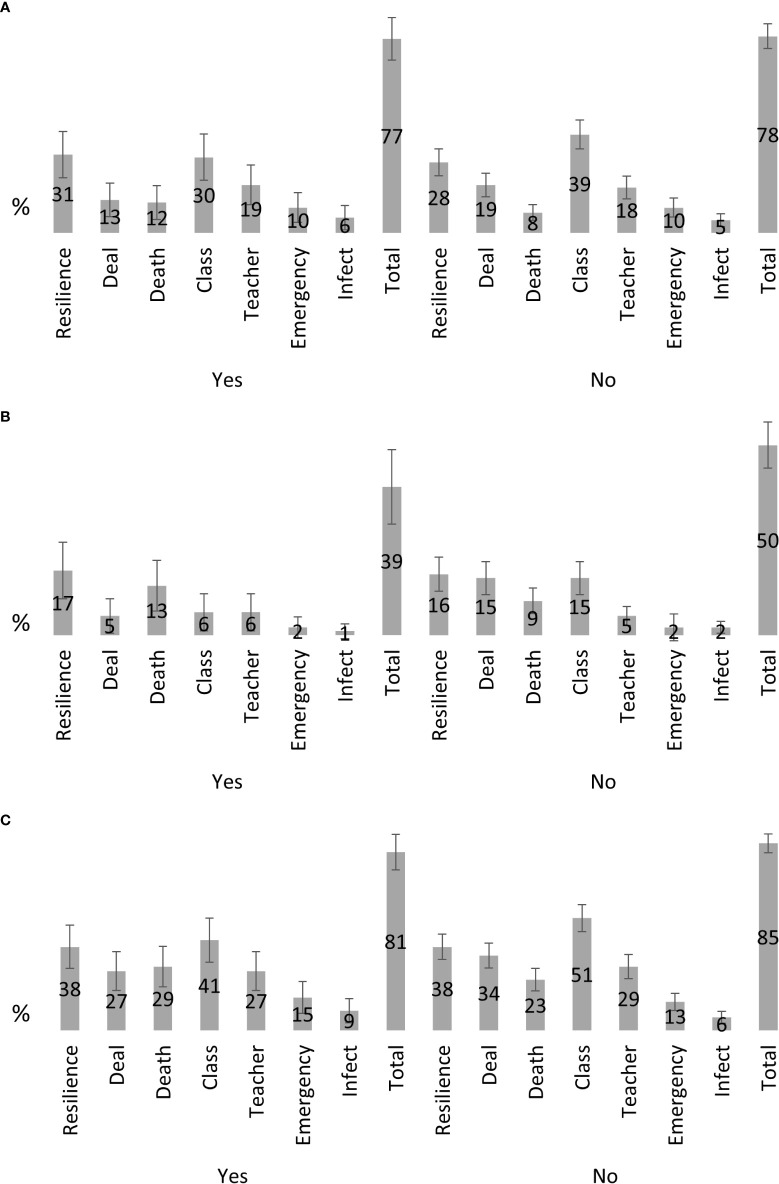
Frequency of concerns reported by teacher (n = 358); **(A)** concerns if a student receives active treatment; **(B)** concerns if a student completed treatment; **(C)** any concerns combined.

Most teachers with (81%) and without (85%) experience reported at least one fear or concern (**Figure 1C**). How to inform the classmates (41% and 51%) and the students’ resilience (38% each) were the most frequently reported concerns in both groups. The topic of death and relapse was reported by a larger proportion of teachers with experience (29%) compared to those without (23%) experience. The topic on how to deal with the student and his or her family was raised more frequent by teachers without experience (34%) compared to those with experience (27%).

Looking at the teachers’ feedbacks having a student under active treatment in their class, 77% of teachers with experience reported at least one concern and 78% of teachers without experience (**Figure 1A**). The three most frequently mentioned concerns in teachers with experience were students’ resilience (31%), how to deal with classmates (30%) and the teachers’ personal concerns (19%). Teachers without experience reported most frequently concerns about classmates (39%), students’ resilience (28%) and how to deal with the student or his or her family (19%).

Looking at the teachers’ feedbacks having a student in the class who completed the treatment already, the distribution of each of the concerns is similar to teachers who have a student under active treatment, but the proportions of each concern are lower (**Figure 1B**). Only 39% of teachers with experience report any concern and 50% of teachers without experience. Again, students’ resilience and the topic of death and relapse were reported most frequently.

Most teachers consider it important to inform the classmates having a child or adolescent with cancer (85%, **Table 4**). Most teachers prefer to be involved informing the classmates (76%), 33% prefer the involvement of physicians and one fourth (26%) a written document (**Table 4**). More than 80% of teachers prefer to inform the classmates about what the treatment means for the affected child or adolescent and how they can participate in school lessons and sports. Half of the teachers (**Table 4**) prefer information about cancer in general (e.g. the meaning of the word “cancer”, different types of cancer and cancer treatment).

**Table 4:** Teachers’ perception if/how to inform classmates (n=358).

	Number
**Is it important to inform the classmates?** Yes No Missing	307 (86) 37 (10) 14 (4)
**How should classmates be informed?** Written information/document Physician Teacher Other*	94 (26) 118 (33) 274 (76) 155 (43)
**Which information should classmates receive?** Cancer general Cancer treatment What treatment means for the affected child Participation school, sports Death Other	191 (53) 194 (54) 320 (89) 304 (85) 172 (48) 55 (15)

*most statements include individual approach and depending on the patients/parents preferences; child/adolescent himself if possible [n=43]; parents [n=17]; parents and/or child/adolescent [n=21]; social worker or psychologist from school [n=7]; video or books [n=4]; hospital tour [n=2].

The topics mentioned in this survey resulted in the development of a short flyer. The flyer covers the following topics (one page each): 1) childhood cancer in Switzerland in general, 2) a child or adolescent receiving active treatment, 3) a child or adolescent, who already completed treatment, 4) dealing in everyday school life, 5) contact details and further literature and information (websites and books about the topic).

## Discussion

Our results show, that over 80% of teachers reported fears, concerns and uncertainties, independent of whether they had experience with a student diagnosed with cancer or not. The topics reported most frequently did not differ between the situation of a child or adolescent newly diagnosed with cancer compared to a cancer survivor. Our results underline, that there is an information need among most teachers how to handle these new situations appropriately.

The concerns reported by teachers from our study are comparable to the information needs of teachers reported by Brown et al. (10) and Chekryn et al. (11). Brown et al. assessed the needs of 528 teachers to develop a computer-based training program. One section asked the teachers to state areas where additional information would be helpful. Physical limitations (89%), talking to the child (84%) or the child’s classmates (84%), and the emotional impact on the families (76%) were reported most frequently (10). These areas were also considered important by teachers from our survey. Chekryn et al. interviewed nine children, parents and teachers 4-6 weeks following the return to school. Uncertainties were related to academic expectations, information needs on the disease and its course versus the patients right for privacy, how to talk about emotions, and the personal impact on the teachers, including the feeling of being unprepared (11). Even though Chekryn et al. published their results in 1987, the topics are still comparable to those mentioned in our survey. This is supported by the results from Fryer et al. published in 1989 (12). Their survey was answered by 33 teachers, who reported becoming too emotional, especially explaining to the class a student’s death, as the main concerns. The publication by Chekryn et al. also highlighted the important aspect of privacy (11). Nowadays, it is considered obvious, that any information about the child or adolescent can only be given with his or her consent or the parent’s consent. This privacy must be respected and is defined differently and individually by each child, adolescent or their parents.

In 2002, Papadatou et al. performed a survey to explore the experiences of Greek teachers regarding the school integration of children and adolescents diagnosed with a chronic or life-limiting condition (13). From the 1792 respondents, 19% (n=340) reported having experience with a chronically sick child. Cancer was the second most frequent condition reported by the teachers with experience. Asked about situations, that affected them most, they reported most frequently 1) the inability to handle emergencies, 2) the child’s physical changes and 3) the child’s strategies to cope with the illness (13). Participants from our survey also emphasized the first two topics. Two third (60%) of Greek teachers recognized changes on communication and behavior towards the sick child. They avoided pressure and criticism, had fewer expectations for academic performance and showed greater support. Teachers, who did not report changes in their attitudes, tried not to differentiate between the sick child and the classmates. This dichotomy was also reported by the participants form our survey. The perception of the Greek teachers might not directly be comparable to our cohort as childhood cancer treatment is often an intense phase of a given duration, often followed by a slowly returning normality. This is often not the case for other chronic diseases, such as sickle cell anemia, epilepsy, paralysis or diabetes, which persist life-long. These differences in anticipated duration of the disease time might influence the teachers’ attitudes towards the sick child, but also the current situation of the health status and the prognosis. These factors again influence the teachers’ potential fears and uncertainties.

Different school reintegration programs are described today, and teachers and school staff are involved to different degrees (8). Thompson et al. conclude that these programs are very much appreciated by patients, parents and school staff. They contribute to a better understanding among teachers and classmates regarding the disease and its consequences for the sick child (8). School reintegration programs might be the right place to provide information about the topics raised in our survey.

Annett et al. performed a feasibility study and evaluated a school reintegration intervention for children diagnosed with acute lymphoblastic leukemia. The intervention lasted over 4 months and was performed by a family advocate (7). The intervention consisted of eight consecutive modules: 1) getting to know you and your child, 2) communication between family, school and hospital, 3) communication with school personnel, 4) treatment effects upon the sibling(s), 5) advocacy, 6) short-term effects of acute lymphoblastic leukemia treatment 7) long-term effects of acute lymphoblastic leukemia treatment, 8) closure/review. The authors conclude, that the intervention proved to be satisfactory in the eight participating families (7). However, all families were off treatment already, and the perspective of teachers’ fears and concerns seems not to be part of any module. From a teachers’ perspective and based on our results, we advocate on their early involvement. A first module could for instance achieve this at the time of cancer diagnosis. In addition, addressing potential fears and concerns proactively by the health care providers can give the teachers a trustful relationship and influence the future collaboration positively. This also gives the teachers an opportunity to address topics, that they may not dare to address spontaneously, such as being afraid to be too emotional or to be unsure on how to deal with the child and his/her parents when they meet the first time following the diagnosis. Several publications highlighted that information is a crucial element to reduce the fears and uncertainties of teachers having a child with cancer or another long-term health condition in their class (10, 14, 15). In 1992, Katz et al. evaluated a school reintegration intervention, conducted by hospital-based pediatric psychologists (16). The intervention had the following five components: 1) preventive education including the information of teachers and school personnel by phone, 2) identification of a hospital-based liaison, 3) preparation of the child’s return to school, 4) informative presentations to school personnel and classmates, 5) periodic follow-up. The fourth component is targeted to the teachers’ needs and to inform them adequately. This might be the right place again to proactively address the fears and concerns mentioned in our survey.

In 1988, Stevens et al. assessed, how hospitals in the United Kingdom liaised with the schools of a child diagnosed with cancer (17). They explicitly asked about the provision of written information. Five out of 13 participating hospitals used written documents specifically designed for the teachers. The documents combined covered the following five topics: overview of childhood cancer, risk of infection and medical problems at school, children’s knowledge of their disease, school attendance and academic performance (17). These topics are also covered in our flyer. This highlights, that even though the field of pediatric oncology and school system changed a lot since 1988, the main information needs remained the same.

## Limitations and strength

Approaching the principals from elementary schools in the canton of Aargau through the cantonal authority only and searching the remaining schools on public websites may have resulted in a participation bias. It made sending-out reminders impossible. Through this approach, we were also not able to calculate the response rate. A personalized mail to each teacher may have increased the return of completed questionnaires. Even though we tried to locate all schools in the catchment area, we might have missed some. Questionnaire-based studies have the inevitably risk of participation bias, resulting in a selected population. It might be that, more anxious teachers participated in the survey or those, who already had a childhood cancer patient in their class, but did not receive enough information. We did not collect detailed information from teachers, who received information to assess whether the needs changed over time. It was statistically not possible to compare teachers, who received information versus those without as the difference in the number of teachers was too big. Despite these limitations, the strengths of this study are the participation of teachers from all different school levels and the relatively large sample size. Therefore, we think that the results are generalizable for all teachers in Switzerland and even for other countries with similar healthcare and educational systems. Evaluating the impact of the flyer was not part of this study and has to be assessed prospectively.

## Conclusion

Teachers do have specific needs having a childhood cancer patient or survivor in their class. Knowledge of these needs is crucial to address them appropriately and to facilitate school reintegration of the affected child or adolescent. A flyer, such as the one developed within this study, covering topics common for all childhood cancer patients and survivors, may be a first guidance, but has to be evaluated in a future step. However, from the setup and clinical experience, such a flyer cannot replace an individual approach for each childhood cancer patient or survivor, neither the direct communication between patients, parents, health care professionals and teachers.

## Data availability statement

The raw data supporting the conclusions of this article will be made available by the authors, without undue reservation.

## Ethics statement

Ethical review and approval was not required for the study on human participants in accordance with the local legislation and institutional requirements. Written informed consent for participation was not required for this study in accordance with the national legislation and the institutional requirements.

## Author contributions

Conceptualization, KS and MO. Data collection, MO. Formal analysis, MO. Funding acquisition, MO. Writing - original draft, MO. Writing - review and editing, KS and MO. All authors contributed to the article and approved the submitted version.

## Funding

Research Council (Forschungsrat) Kantonsspital Aarau.

## Conflict of interest

The authors declare that the research was conducted in the absence of any commercial or financial relationships that could be construed as a potential conflict of interest.

## Publisher’s note

All claims expressed in this article are solely those of the authors and do not necessarily represent those of their affiliated organizations, or those of the publisher, the editors and the reviewers. Any product that may be evaluated in this article, or claim that may be made by its manufacturer, is not guaranteed or endorsed by the publisher.

## References

[1] Galobardes B Shaw M Lawlor DA Lynch JW Davey Smith G . Indicators of socioeconomic position (part 1). J Epidemiol Community Health (2006) 60(1):7–12. doi: 10.1136/jech.2004.023531 PMC2465546 16361448

[2] White IR Blane D Morris JN Mourouga P . Educational attainment, deprivation-affluence and self reported health in Britain: a cross sectional study. J Epidemiol Community Health (1999) 53(9):535–41. doi: 10.1136/jech.53.9.535 PMC1756962 10562877

[3] Tsimicalis A Genest L Stevens B Ungar WJ Barr R . The impact of a childhood cancer diagnosis on the children and siblings’ school attendance, performance, and activities: A qualitative descriptive study. J Pediatr Oncol Nurs (2018) 35(2):118–31. doi: 10.1177/1043454217741875 29192538

[4] Vance YH Eiser C . The school experience of the child with cancer. Child: care Health Dev (2002) 28(1):5–19. doi: 10.1046/j.1365-2214.2002.00227.x 11856182

[5] An H Lee S . Difficulty in returning to school among adolescent leukemia survivors: A qualitative descriptive study. Eur J Oncol Nursing (2019) 38:70–5. doi: 10.1016/j.ejon.2018.12.008 30717939

[6] Elsbernd A Pedersen KJ Boisen KA Midtgaard J Larsen HB . On your own”: Adolescent and young adult cancer survivors’ experience of managing return to secondary or higher education in Denmark. J Adolesc young adult Oncol (2018) 7(5):618–25. doi: 10.1089/jayao.2018.0058 29985720

[7] Annett RD Erickson SJ . Feasibility of a school reintegration programme for children with acute lymphoblastic leukaemia. Eur J Cancer Care (2009) 18(4):421–8. doi: 10.1111/j.1365-2354.2009.01128.x 19594612

[8] Thompson AL Christiansen HL Elam M Hoag J Irwin MK Pao M . Academic continuity and school reentry support as a standard of care in pediatric oncology. Pediatr Blood cancer (2015) 62 Suppl 5:S805–17. doi: 10.1002/pbc.25760 PMC5198902 26700927

[9] Tresman R Brown M Fraser F Skinner R Bailey S . A school passport as part of a protocol to assist educational reintegration after medulloblastoma treatment in childhood. Pediatr Blood cancer (2016) 63(9):1636–42. doi: 10.1002/pbc.26071 27196034

[10] Brown MB Bolen LM Brinkman TM Carreira K Cole S . A collaborative strategy with medical providers to improve training for teachers of children with cancer. J Educ psychol Consultation. (2011) 21:2:149–65. doi: 10.1080/10474412.2011.571478

[11] Chekryn J Deegan M Reid J . Impact on teachers when a child with cancer returns to school. Children’s Health Care (1987) 15:3:161–5. doi: 10.1080/02739618709514764

[12] Fryer LL Saylor CF Finch AJ Jr. Smith KE . Helping the child with cancer: what school personnel want to know. Psychol Rep (1989) 65(2):563–6. doi: 10.2466/pr0.1989.65.2.563 2798671

[13] Papadatou D Metallinou O Hatzichristou C,LP . Children with chronic and life-limiting conditions: Teachers’ perceptions and experiences regarding students’school reintegration. Illn Crisis Loss (2022) 10(2):108–24. doi: 10.1177/105413730201000202

[14] Vanclooster S Benoot C Bilsen J Peremans L Jansen A . Stakeholders’ perspectives on communication and collaboration following school reintegration of a seriously ill child: A literature review. Child Youth Care Forum (2018) 47(4):583–612. doi: 10.1007/s10566-018-9443-4

[15] Hinton D Kirk S . Teachers’ perspectives of supporting pupils with long-term health conditions in mainstream schools: a narrative review of the literature. Health Soc Care Community (2015) 23(2):107–20. doi: 10.1111/hsc.12104 24666555

[16] Katz ER Varni JW Rubenstein CL Blew A Hubert N . Teacher, parent, and child evaluative ratings of a school reintegration intervention for children with newly diagnosed cancer. Children’s Health Care (1992) 21(2):69–75. doi: 10.1207/s15326888chc2102_1 10117965

[17] Stevens MC Kaye JI Kenwood CF Mann JR . Facts for teachers of children with cancer. Arch Dis childhood (1988) 63(4):456–8. doi: 10.1136/adc.63.4.456 PMC1778826 3365019

