# Supplementary material for: Back to school – The teachers’ worries and needs having a childhood cancer patient or survivor in their class
Source: Front Oncol. 2022 Nov 2;12:992584. doi: 10.3389/fonc.2022.992584 (PMC9667046; doi:10.3389/fonc.2022.992584)
Supplement: Supplementary file 1 [file DataSheet_1.docx]

Supplementary Material

*Back to School – The Teachers’ Worries and Needs having a Childhood Cancer Patient or Survivor in their Class*

**Supplemental Explanation E1:** Survey

| **General** | | |
| --- | --- | --- |
| 1 | Sex | Male, female |
| 2 | Current age | ___ [years] |
| 3 | Years working as a teacher | ___ [years] |
| 4 | School level | Primary level   - Nursey - Primary school   Secondary level I   - Secondary school - District school   Secondary level II   - Basic vocational training - Secondary business school - Computer science high school - Professional baccalauteate - High school |
| 5 | Pensum | - Class teacher - Single school subjects |
| 6 | School subject | [free text] |
| 7 | Region | - Urban - Rural |
| **Explanation**: We distinguish between children/adolescents who are newly diagnosed with cancer or are receiving therapy and children/adolescents whose cancer therapy has been completed. | | |
| **Child/ adolescent with cancer** | | |
| 8 | Have you had a student with cancer in your class? | - Yes - No |
| 8a | **If yes,** what situation was the student in? | - Newly diagnosed / receiving treatment - Cancer treatment completed |
| 8b | **If yes, h**ave you received information about cancer in children / adolescents? (e.g. short description, handling of the child) | - Yes - No |
|  | - **If yes**, where have you received the information from? | - Hospital - Parents - Other [free text] |
|  | - **If yes,** in what format did you receive the information? | - Information sheet from the hospital - Information from treating physicians - Parents - Link for a homepage - Other [free text] - Comment [free text] |
|  | - **If yes**, what kind of information did you receive? | - Information on cancer in children/adolescents in general - Information on specific cancer of the child/adolescent - Accessibility in case of emergency - Information on possible emergency situations - Information on resilience of the student (physical/mental) - Other [free text] |
|  | - **If yes**, was the format appropriate? | - Yes - No |
|  | - **If yes,** Was the information sufficient? | - Yes - No |
|  | - **If no,** which information was missing? | [free text] |
|  | - **If no,** what format would you have liked? | [free text] |
| 8c | **If no, w**hat information would you like to see? | [free text] |
| 8d | **If no**, how would you like to receive the information? | - Flyer / general information brochure - Direct information from physician - Parents - Other [free text] |
| 8d | **If no,** which information would be important for you? | - Information on cancer in children/adolescents in general - Information on specific cancer of the child/adolescent - Accessibility, e.g. of the hospital - Information on possible emergency situations - Information on resilience of the student (physical/mental) |
| 8e | Is there anything you have respect for when you imagine a student diagnosed with cancer coming to your class tomorrow who is still on therapy? | [free text] |
| 8f | Is there anything you have respect for when you imagine a student diagnosed with cancer coming to your class tomorrow who has completed therapy? | [free text] |
| 8g | Is there anything else you have respect for? | [free text] |
| **Classmates**  **Important:** The information of classmates always takes place only after consultation with the child/adolescent concerned and his/her parents. The same applies to the extent of the information. | | |
| 9 | Is it important for you to inform the classmates? | - Yes - No |
| 10 | How should classmates be informed? | - Age-appropriate written documents - Verbal information by hospital staff (e.g. doctor) - By teacher - Other [free text] |
| 11 | What information might be important for classmates? | - General information about cancer - What cancer treatment entails - What cancer treatment means for the affected child/adolescent - Impact on participation in school, sports, seeing friends, etc. - The topic of death |
| **Closing** | | |
| 12 | Would an information brochure be helpful from your point of view?  (adapted to school levels) | - Yes - No |
| 12a | - **If yes,** what should the information brochure contain? | [free text] |
| 12b | - **If no,** why is this not helpful from your point of view | [free text] |

**Table S1:** Comparison of teacher characteristics who completed and did not complete the survey completely

|  | All data available (n=358) | Only characteristics available  (n=59) | p-value |
| --- | --- | --- | --- |
|  | Number (%) | Number (%) |  |
| **Sex**  Female | 288 (80) | 42 (71) | 0.268 |
| **Age**  18 – 24 years  25 – 34 years  35 – 44 years  45 – 54 years  55 – 64 years  65 years or older | 10 (3)  73 (20)  81 (23)  100 (28)  90 (25)  4 (1) | 0 (0)  17 (29)  19 (32)  8 (14)  14 (24)  1 (2) | 0.092 |
| **Working years** [years] Median (IQR) | 18 (8 - 25) | 15 (8 – 20) | 0.215 |
| **School level**  Primary level   - Nursey - Primary school   Secondary level I   - Secondary school - District school   Secondary level II   - Basic vocational training - Secondary business school - Computer science high school - Professional baccalauteate - High school | 67 (19)  159 (44)  68 (19)  32 (9)  39 (10)  23 (6)  14 (4)  13 (4)  58 (16) | 11 (19)  26 (44)  11 (19)  4 (7)  7 (12)  2 (4)  0 (0)  1 (2)  9 (15) |  |
| **Pensum**  Class teacher  Individual school subjects  Other | 181 (51)  119 (33)  58 (16) | 34 (58)  17 (29)  8 (14) | 0.601 |
| **Region**  Rural  Urban | 192 (54)  166 (46) | 33 (56)  26 (44) | 0.742 |
| **Experience**  Yes  No | 94 (26)  264 (74) |  |  |
| **Brochure helpful**  Yes  No  Missing | 264 (74)  77 (21)  17 (5) |  |  |
